# Pneumococcal carriage and serotype distribution in children with nephrotic syndrome

**DOI:** 10.1007/s00467-024-06423-4

**Published:** 2024-06-05

**Authors:** Tugba Erem, Asli Kavaz Tufan, Omer Kilic, Aysun Caltik Yilmaz, Yalcın Kara, Mahmut Can Kizil, Meltem Dinleyici, Nuran Cetin, Mucahit Kaya, Ener Cagri Dinleyici

**Affiliations:** 1 Department of Pediatrics, Faculty of Medicine, Eskisehir Osmangazi University, Eskisehir, TR-26040 Turkey; 2 Department of Pediatric Nephrology, Faculty of Medicine, Eskisehir Osmangazi University, Eskisehir, Turkey; 3 Department of Pediatric Infectious Diseases, Faculty of Medicine, Eskisehir Osmangazi University, Eskisehir, Turkey; 4 Department of Pediatric Nephrology, Ankara Etlik City Hospital, Ankara, Turkey; 5 Department of Social Pediatrics, Faculty of Medicine, Eskisehir Osmangazi University, Eskisehir, Turkey; 6 Diagen Biotechnology, Ankara, Turkey

**Keywords:** Nephrotic syndrome, Pneumococci, Carriage, Pneumococcal vaccines

## Abstract

**Background:**

Patients with nephrotic syndrome (NS) are at a higher risk of developing invasive pneumococcal disease (IPD). Pneumococcal carriage studies are helpful tools for detecting potentially infectious serotypes and guiding immunization efforts. Pneumococcal nasopharyngeal colonization is common, and IPD can easily occur in an immunosuppressed state. Limited information is available regarding the frequency of pneumococcal carriage in individuals with NS. The aim of this study was to evaluate pneumococcal carriage and serotype distribution in children with NS.

**Methods:**

Pneumococcal carriage was detected by real-time PCR assays from nasopharyngeal swab samples from 98 children with NS, and 100 healthy controls. Isolates were serotyped by real-time PCR.

**Results:**

The pneumococcal carriage rate was 44.9% in children with NS. Regarding the recommendation about pneumococcal immunization in children with NS, the vaccination rate was low. Also, non-PCV13 serotypes have been detected in at least 25% of PCV13-vaccinated children. There is no statistically significant difference in total pneumococcal carriage rate, PCV13 serotype carriage rate, or non-PCV13 serotype carriage rate between children with NS and healthy controls (*p* > 0.05 for all).

**Conclusions:**

The pneumococcal carriage rate was similar between children with NS and healthy controls. However, because children with NS have an increased risk for IPD, the serotype distribution of children with NS can demonstrate the improved protection offered by new pneumococcal vaccines. Regular monitoring for IPD is crucial for assessing the evolving sero-epidemiology of pneumococcal infections and evaluating the effectiveness of vaccines for children with NS.

**Graphical Abstract:**

**Figure:**
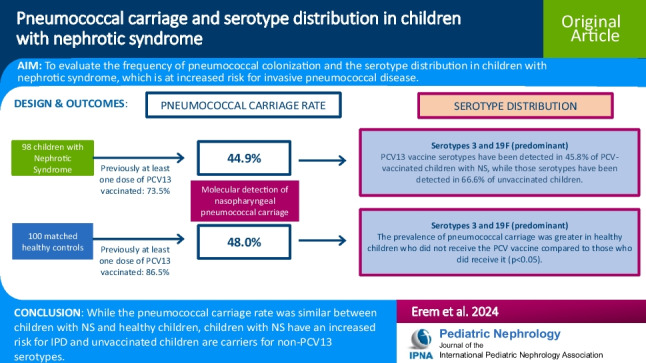
A higher resolution version of the Graphical abstract is available as Supplementary information

**Supplementary Information:**

The online version contains supplementary material available at 10.1007/s00467-024-06423-4.

## Introduction

*Streptococcus pneumoniae* is an important and common cause of upper respiratory tract infections, pneumonia, meningitis, bacteremia, and sepsis in both children and adults worldwide [1]. Invasive pneumococcal disease (IPD) is higher in children below the age of two and individuals aged 65 and above [2]. People with functional and anatomical asplenia, sickle cell anemia, nephrotic syndrome, human immunodeficiency virus, cochlear implants, cancer, cerebrospinal fluid leaks, and long-term heart and/or lung disorders have an increased risk for IPD [3]. Vaccines developed using capsule polysaccharides (polysaccharide or conjugated) are the most effective method for protecting children and adults from pneumococcal infections [2, 3]. The widespread use of conjugated pneumococcal vaccines (PCVs) in routine immunization programs has led to a notable reduction in the occurrence of IPD in both infants and adults, as well as mucosal infections caused by the pneumococcal strains targeted by the vaccines. Nevertheless, the incidence of infection caused by non-vaccine serotypes has increased, even if there has been a decline in the overall prevalence of IPD because of vaccination [4].

Nephrotic syndrome (NS) is a common and significant kidney disorder in children. People with NS have an immunodeficiency due to lymphocyte dysfunction, T cell dysregulation during relapse and remission, low levels of albumin leading to edema, impaired spleen function, kidney loss of immunoglobulins, complements, and other proteins, and the use of immunosuppressant drugs [5]. Each year, around 1–2% of patients with NS suffer from invasive bacterial infections, specifically pneumococcal infections including peritonitis, bacteremia, and pneumonia and sepsis continues to be a leading cause of death in children with NS [5–8]. Despite the availability of PCVs, children with high-risk conditions remain vulnerable to IPD [9]. Van Warmerdam et al. [9] retrospectively evaluated IPD in high-risk children between 2000 and 2018 in Canada. Among the 94 hospitalized high-risk patients with IPD, 9% of children had NS and all had bacteremia. Matthew et al. [7] recently reported 63 isolates of pneumococci obtained from 60 children with NS, between 2007 and 2021, which represented 18% of all pneumococcal infections occurring in children during the same period in India. PCV10 and PCV13 covered up to 58% of all the serotypes causing infection. Severe disease, with shock, intensive care admission, and/or meningitis, was observed in 38% of children, and mortality was observed in 10%.

Pneumococcal vaccines are essential and routinely recommended for preventing life-threatening pneumococcal infections in children with NS [5]. *Streptococcus pneumoniae* can colonize the nasopharynx without any symptoms, especially in children, and spread to nearby mucosal tissues or get into the bloodstream [1]. Invasive infections can easily occur during an immunosuppressed state like NS [7]. Regularly monitoring the sero-epidemiology of pneumococcal infections is recommended when there are changes in the serotype epidemiology over time and across different geographical areas [10]. There are limited data about the pneumococcal carriage in patients with NS. The aim of this study was to evaluate the frequency of pneumococcal colonization and the distribution of serotypes in children with NS and to compare with children without NS.

## Patient and methods

The objective of this study was to evaluate the frequency and serotype distribution of nasopharyngeal pneumococcal colonization in children with NS. Patients followed up for NS at the Eskisehir Osmangazi University Faculty of Medicine and the Department of Pediatric Nephrology at Baskent University Hospital were included in the study between April and August 2022. We selected healthy children as the control group from those who applied to the outpatient clinic for routine follow-up during the same enrollment period as children with NS. The project received approval from the Eskisehir Osmangazi University Clinical Research Ethics Committee (January 18, 2022; decision number 37). Written informed consent to participate in this study from parents and the children themselves, if they are above 11, has been received.

We obtained age, gender, age at diagnosis, pathologic diagnosis, use of immunosuppressive agents, and presence of pneumococcal vaccines from the patients’ medical records. The National Immunization Program (NIP) in Turkey incorporated the PCV7 vaccine in 2008 and replaced it with PCV13 in 2011. PCV13 and PPSV23 are also administered to those in high-risk groups, including NS.

A sterile cotton swab was inserted through one side of the nose, and the nasopharynx was sampled. Following the collection of nasopharyngeal swab samples, they were immersed in DiaRex® Glycerol Buffered Saline (Diagen Biotechnology, Ankara, Turkey) and subsequently stored at a temperature of − 80 °C. The DNA was extracted using the DiaRex® DNA extraction kit (BLD-5295, Diagen Biotechnology, Ankara, Turkiye) and subsequently tested for the presence of *Neisseria meningitidis*, *Streptococcus pneumoniae*, and *Haemophilus influenzae* using the ECD_DNZ 2010 Realtime PCR Kit (Diagen Biotechnology, Ankara, Turkey) on the BioradCFX96 platform. Samples were considered positive for the presence of the targeted sequence when the serotype/serogroup specific signal was ≤ 35 cycle threshold. The samples that tested positive for *S. pneumoniae* were subjected to Sanger sequencing and examined using the methodology described by Marmaras et al. [11]. The sequences were subsequently uploaded to the *S. pneumoniae* CST Typing Tool to determine the serotype of *S. pneumoniae*  [12].

PCV13 covered serotypes were as follows: 1, 3, 4, 5, 6A, 6B, 7F, 9 V, 14, 18C, 19 A, 19F, and 23F. Pneumococcal isolates were classified as PCV13 serotypes (1, 3, 4, 5, 14, 19A, 19F, and 23F), those belonging to serogroups with both PCV13 and non-PCV13 serotypes (6 A/B/C/D, 7A/F, 9 A/L/N/V, 18 C/F), and non-PCV13 serotypes (remaining serotypes) [13].

A statistical analysis was performed with the Statistical Package for the Social Sciences (SPSS, Chicago, IL, USA). Non-normally distributed data were shown as medians (interquartile range, IQR). A non-parametric Mann–Whitney *U* test was performed to compare results between groups for non-normally distributed data. Fisher’s exact test or chi-square tests of association were applied to assess whether any differences existed between each categorical factor. A *p*-value of < 0.05 was used to determine statistical significance.

## Results

There were 98 children with NS (28 girls and 70 boys) between the ages of 3 and 17 years, with a median age of 10.5 years. The healthy control group consisted of 100 children (50 boys and 50 girls) between the ages of 2 and 17 years, with a median age of 9 years. Boys outnumbered girls in the NS group. There is no difference in age between the NS group and controls (*p* > 0.05). Regarding clinical and histological findings of children with NS, 64 (65.3%) had minimal change disease (MCD). All MCD cases are presumed, and no biopsy results are available. The clinical pathological diagnosis of the remaining 34 cases is summarized in Table 1. A total of 76 (77.5%) children with NS were in remission, while 22 (22.5%) were experiencing a relapse. Additionally, 35 (35.7%) of the patients were currently undergoing treatment with immunosuppressive medications at the time of data collection.

**Table 1:** Demographic, clinical, and immunization status of children with nephrotic syndrome

	Nephrotic syndrome *n* = 98	Control *n* = 100
Age (years)*	10.5 (8)	9 (8)
Boys/girls	70/28	50/50
Cause of nephrotic syndrome		
Minimal change disease *n* (%)	64 (65.3)	
Focal segmental glomerulosclerosis *n* (%)	14 (13.3)	
Membranoproliferative glomerulonephritis *n* (%)	9 (8.6%)	
Mesangial proliferative glomerulonephritis *n* (%)	4 (3.8%)	
Congenital nephrotic syndrome *n* (%)	2 (1.9%)	
Membranous nephropathy *n* (%)	2 (1.9%)	
NS due to COQ6 deficiency *n* (%)	1 (1%)	
Diffuse mesangial sclerosis *n* (%)	1 (1%)	
Focal segmental glomerulosclerosis and IgM nephropathy *n* (%)	1 (1%)	
Remission	76 (77.5%)	
Relapse	22 (22.5%)	
Current immunosuppressive medications	35 (35.7%)	
Time since the diagnosis of NS (years)*	3.5 (4)	
PCV immunization status		
PCV13 only (3 plus one dose)	57 (58.2%)	67 (67%)
PCV13 (3 plus one dose) and PPSV23 (one dose)	6 (6.1%)	-
Partially vaccinated with PCV13 (lower than 4 doses)	9 (9.2%)	19 (19%)
Unvaccinated	26 (26.5%)	14 (14%)

*NS*, nephrotic syndrome; *PCV*, pneumococcal conjugated vaccine; *PCV13*, 13-valent conjugated pneumococcal vaccine; *PPSV23*, 23-valent pneumococcal polysaccharide vaccine * median (IQR)

The assessment of the pneumococcal vaccination status of children with NS is shown in Table 1. Immunization records showed that 72 children with NS received at least one dose of PCV13 (57 children received four doses (3 plus one dose) PCV13, six children received four doses of PCV, and one dose of PPSV23; and nine children received PCV13 lower than the recommended four doses). Twenty-six children with NS did not receive PCV.

The NS group had a pneumococcal carriage prevalence of 44.9% (*n* = 44), and the most common serotypes are 3 and 19F (Tables 2 and 3). The groups vaccinated with the PCV vaccine and those who were not vaccinated showed no statistically significant difference in pneumococcal carriage (*p* > 0.05). PCV13 vaccine serotypes have been detected in 45.8% of PCV-Pneumococcal Serotype Identification by Capsular Sequencevaccinated children with NS, while they have been detected in 66.6% of unvaccinated children. Regarding the presence of PCV13 serotypes, the nasopharyngeal carriage rate was as follows in PCV13 vaccinated children with NS: PCV13 serotypes (50%), those belonging to serogroups with both PCV13 and non-PCV13 serotypes (25%), and non-PCV13 serotypes (25%). Two children who were previously vaccinated with PCV13 plus PPSV23 have a pneumococcal carriage, and both are non-PCV 13 serotypes (serotypes 10A/B and 11A). The serotype distribution of unvaccinated children with NS was as follows: PCV13 serotypes (66.6%), those belonging to serogroups with both PCV13 and non-PCV13 serotypes (16.7%), and non-PCV13 serotypes (33.3%). In the NS group, we found pneumococcal carriage in 21.5% of the 22 relapsed cases and in 47.3% of the 76 remission cases (Tables 2 and 3).

**Table 2:** Pneumococcal carriage isolated serotype distribution according to their inclusion in the PCV13 in NS and healthy control groups

	PCV13 serotypes	Serogroups with both PCV13 and non-PCV13	Non-PCV13	Total
Nephrotic syndrome	22 (50%)	11 (25%)	11 (25%)	44/98 (44.9%)
PCV13 vaccinated	14 (43.8%)	9 (28.1%)	9 (28.1%)	32/72 (44.4%)
PCV13 only (3 plus one dose)	11 (45.8%)	8 (33.4%)	5 (20.8%)	24/57 (42.1%)
PCV13 (3 plus one dose) and PPSV23 (one dose)	-	-	2 (100%)	2/6 (33.3%)
Partially vaccinated with PCV13 (lower than 4 doses)	3 (50%)	1 (16.7%)	2 (33.3)	6/9 (66.7%)
Unvaccinated	8 (66.6%)	2 (16.7%)	2 (16.7%)	12/26 (46.1%)
Healthy controls	25 (52.0%)	8 (16.7%)	15 (31.3%)	48/100 (48%)
PCV13 only (3 plus one dose)	13 (46.4%)	6 (21.5%)	9 (32.1%)	28/67 (41.8%)
Partially vaccinated with PCV13 (lower than 4 dose)	5 (55.6%)	2 (22.2%)	2 (22.2%)	9/19 (47.3%)
Unvaccinated	7 (63.6%)	-	4 (36.4%)	11/14 (78.5%)

*PCV13*, 13-valent conjugated pneumococcal vaccine; *PPSV23*, 23-valent pneumococcal polysaccharide vaccine. PCV13 covered serotypes were as follows: 1, 3, 4, 5, 6A, 6B, 7F, 9 V, 14, 18C, 19 A, 19F, and 23F. Pneumococcal isolates were classified as PCV13 serotypes, those belonging to serogroups with both PCV13 and non-PCV13 serotypes, and non-PCV13 serotypes

**Table 3:** Serotype distribution of children with NS according to PCV immunization status

	PCV13 SEROTYPES					Serogroups with both PCV13 and non-PCV13				Non-PC13								
	3	14	19A	19F	23F	6 A/B/C/D	7A/F	9 A/L/N/V	18C/F	10A/B	11A	13	15 A/B/C/F	22A/F	23A	33F	35F/37	Not defined
Nephrotic syndrome																		
PCV13 vaccinated	3	1	1	6	3	1		5	3	3	1	1				1		3
PCV13 only (3 plus one dose)	3	1		4	3	1		4	3	2						1		2
PCV13 (3 plus one dose) and PPSV23 (one dose)										1	1							
Partially vaccinated with PCV13 (lower than 4 doses)			1	2				1				1						1
Unvaccinated	6	1		1				2			1							1
Total	9	3	1	7	3	1		7	3	3	2	1				1		4
Healthy controls																		
PCV13 only (3 plus one dose)	7		1	4	1		2	4	1		2		3		1		1	1
Partially vaccinated with PCV13 (lower than 4 dose)	3			2				1					1					2
Unvaccinated	4		1	1	1						1			1		1		1
Total	14		2	7	2		2	5	1		3		4	1	1	1	1	4

*PCV13*, 13-valent conjugated pneumococcal vaccine; *PPSV23*, 23-valent pneumococcal polysaccharide vaccine

Immunization records showed that 86 children (86%) in the control group received at least one dose of PCV13 (67 children received four doses of PCV13; and 19 children received PCV13 lower than four doses, partially vaccinated). Fourteen healthy children did not receive PCV. Nasopharyngeal pneumococcal carriage rate was 48% (*n* = 48), and serotypes 3 and 19F were the most common serotypes. The prevalence of pneumococcal carriage was greater in healthy children who did not receive the PCV vaccine compared to those who did receive it (*p* < 0.05). The serotype distribution of three plus one dose PCV13-vaccinated healthy was as follows: PCV13 serotypes (46.4%), those belonging to serogroups with both PCV13 and non-PCV13 serotypes (21.6%), and non-PCV13 serotypes (32.1%) (Tables 2 and 3).

There is no statistically significant difference in total pneumococcal carriage rate, PCV13 serotype carriage rate or non-PCV13 serotypes carriage rate between children with NS and healthy controls (*p* > 0.05 for all).

## Discussion

In this study, the pneumococcal carriage rate was 44.9% in children with NS. Out of the patients with NS who had not received the pneumococcal vaccines, the pneumococcal carriage rate was found at 46.2%, while it was 50% in unvaccinated children with NS. There is limited information about the pneumococcal carriage rate in patients with NS. Pekuz et al. [14] performed a study in Turkey between 2015 and 2016 about the pneumococcal carriage rate with bacterial culture in 1024 children with chronic disorders, including NS. The study found that the presence of nasopharyngeal carriage in children with chronic disorders was 10.3%, 8% in children with NS, and 8.6% in healthy children [14]. In Poland, Szmigielska et al. [15] demonstrated that the prevalence of pneumococcal carriage in patients with NS was 13.7%, with the most prevalent serotypes being 6B (38.5%), 9 V (15.3%), and 19F (7.6%). The carriage rate of our study is higher than in previous studies. The prevalence of nasopharyngeal carriage and the distribution of different pneumococcal serotypes can vary depending on age, geographical location, daycare attendance, regular use of PCVs, living in crowded environments, and smoking habits [16]. The COVID-19 pandemic influenced pneumococcal infections and sero-epidemiology, like other vaccine-preventable diseases. Another key determinant that sets research apart is whether it employs traditional cultural methods or molecular-based techniques to identify pneumococcal carriage [12]. The higher rates of carriage in our study may be due to use of molecular tests as PCR, compared to lower rates in studies that used culture [14, 15].

In India, according to a study including 364 hospitalized pediatric patients with IPD, a significant proportion of those (17.8%) occurred in children with NS [7]. None of the children with pneumococcal infections had received a dose of any pneumococcal vaccine. A significant number of infections were diagnosed during the first episode of NS and were associated with radiographic features of pneumonia with significant mortality, up to 10% [7]. In this study, the vaccine-preventable serotypes were mainly responsible for IPD and pneumococcal nasopharyngeal carriage in India [7]. For this reason, nasopharyngeal carriage studies would help to understand the IPD sero-epidemiology. The use of molecular diagnostic tools has resulted in significant progress in the identification and serotyping of pneumococcal pathogens. Molecular approaches are crucial in situations where the community frequently uses antibiotics to treat respiratory tract infections, because antibiotic use might affect the culture results [12]. We utilized molecular methodologies to ascertain the identity and serotyping of pneumococci in nasopharyngeal specimens.

The effectiveness of PCV13 against vaccine serotypes in children was found to be 86% [17]. In this study, while routinely recommended, 72 children with NS received at least one dose of PCV13, and only six children received PCV with three plus one dose schedule and one dose of PPSV23. In Canada, despite recommendations to vaccinate high-risk children older than 2 years with PPSV23, only three patients had received PPSV23 among 66 patients with known pneumococcal vaccination status. These data suggest that most children with NS at high risk of IPD are incompletely vaccinated according to standard recommendations [9]. We have detected non-PCV13 serotypes in at least 25% of children with NS. There are new pneumococcal conjugate vaccines (PCV15 includes PCV13 serotypes with serotypes 22F and 33F; PCV 20 includes serotypes 8, 10A, 11A, 12F, 15B, 22F, and 33F), which might provide better and more complete protection [13]. More research needs to be done about the immunogenicity and effectiveness of new PCVs for people with NS. Widespread immunization of the general population and subsequent herd protection are important factors in IPD prevention, especially among high-risk individuals, including patients with NS.

In our study, the prevalence of pneumococcal carriage in the healthy control group was 48%. The incidence of pneumococcal carriage was significantly lower in vaccinated children compared to unvaccinated children. Syrogiannopoulos et al. [12] used molecular techniques, like our study, and found that 48.6% of 1212 Greek children vaccinated against PCV13 also had pneumococcal carriage. In our study, the most common serotypes found in healthy children who received PCV immunization are serotype 3, serotype 19F, serotype 9 V, and serotype 15A/B/C/F. The most common serotype among children who did not get PCV immunization was serotype 3. In Turkey, a recent cross-sectional study about pneumococcal carriage in children was performed on 580 children below 5 years of age, between 2019 and 2020, before the COVID-19 pandemic [18]. The overall carriage rate with standard culture methods was 17.8% among PCV-13 vaccinated children, and the serotype coverage rate was 27.2% for PCV13 [18].

Our investigation has some limitations. The methods adopted in the study evaluated each sample only once, potentially overlooking instances when multiple serotypes may have been present. Additionally, the study did not distinguish between serotypes 6A, 6B, 6C, and 6D; for this reason, we did not exact the coverage rate for new PCVs. In the study group, boys outnumbered girls, and there were differences between the NS group and the control group.

Immunizing children with NS is essential in preventing life-threatening pneumococcal infections. Studies have generally shown pneumococcal vaccination to be safe. It is imperative to increase vaccine coverage to reduce the prevalence of pneumococcal infections in patients with NS. Every pediatric nephrology visit of patients with NS—after the first visit—is an opportunity to screen vaccination status and to administer appropriate vaccines [19]. In this study, the pneumococcal carriage rate was similar between children with NS and healthy children. However, children with NS have an increased risk for IPD compared to healthy children. Conducting examinations of pneumococcal carriage and serotypes, as demonstrated in this work, will also assist in determining vaccination status and identifying new vaccine requirements.

### Supplementary Information

Below is the link to the electronic supplementary material.

Graphical abstract (PPTX 77.0 KB)

## Data Availability

There are no open data.
