# Supplementary material for: Pneumococcal carriage and serotype distribution in children with nephrotic syndrome
Source: Pediatr Nephrol. 2024 Jun 5;39(10):2989–95. doi: 10.1007/s00467-024-06423-4 (PMC11349834; doi:10.1007/s00467-024-06423-4)
Supplement: Supplementary file 1 — Graphical abstract (PPTX 77.0 KB) [file 467_2024_6423_MOESM1_ESM.pptx]

## Slide 1
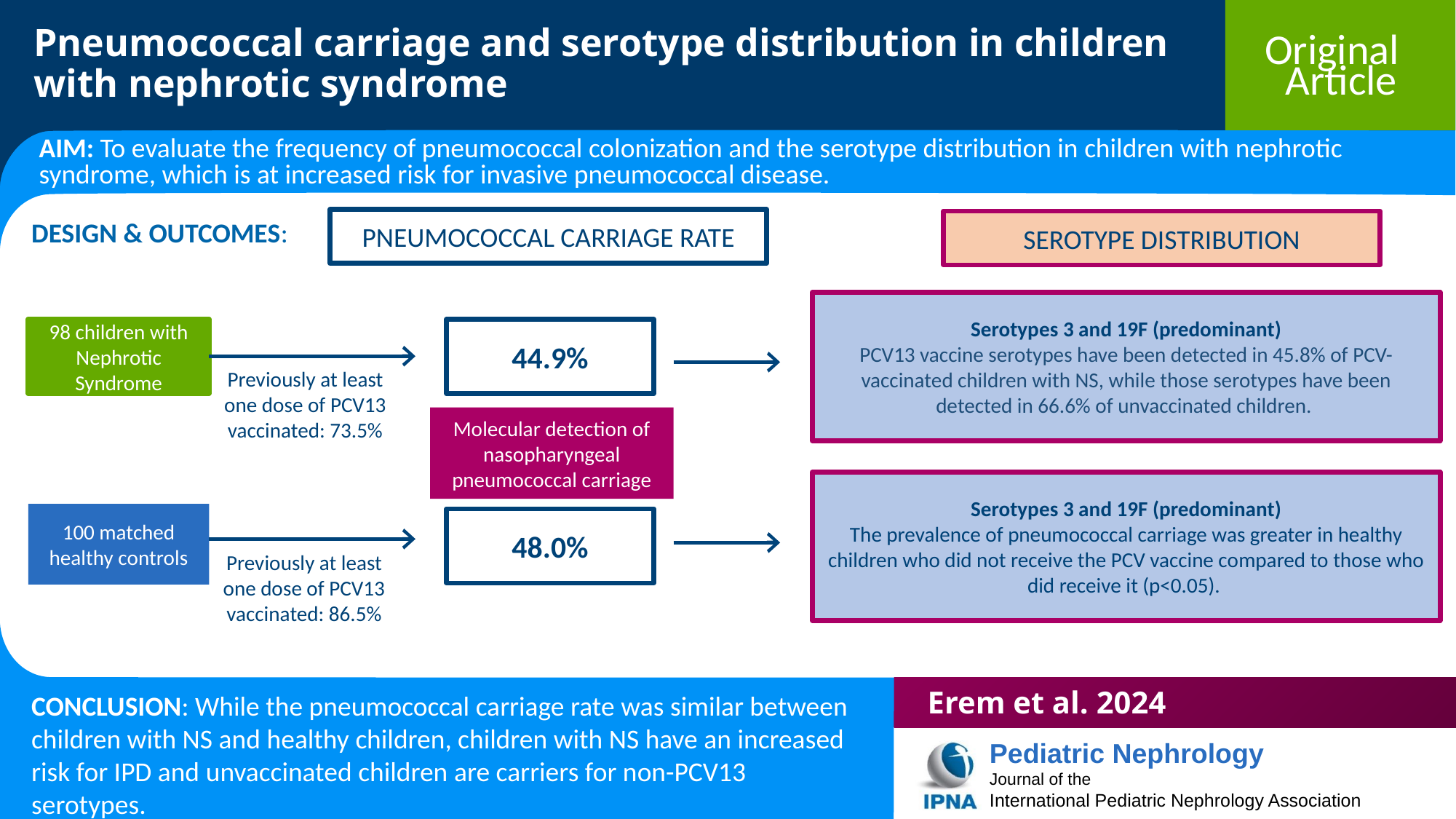

Pneumococcal carriage and serotype distribution in children
with nephrotic syndrome
AIM: To evaluate the frequency of pneumococcal colonization and the serotype distribution in children with nephrotic syndrome, which is at increased risk for invasive pneumococcal disease.
PNEUMOCOCCAL CARRIAGE RATE
DESIGN & OUTCOMES:
SEROTYPE DISTRIBUTION
Serotypes 3 and 19F (predominant)
PCV13 vaccine serotypes have been detected in 45.8% of PCV-vaccinated children with NS, while those serotypes have been detected in 66.6% of unvaccinated children.
98 children with Nephrotic Syndrome
44.9%
Previously at least one dose of PCV13 vaccinated: 73.5%
Molecular detection of nasopharyngeal pneumococcal carriage
Serotypes 3 and 19F (predominant)
The prevalence of pneumococcal carriage was greater in healthy children who did not receive the PCV vaccine compared to those who did receive it (p<0.05).
100 matched healthy controls
48.0%
Previously at least one dose of PCV13 vaccinated: 86.5%
Erem et al. 2024
CONCLUSION: While the pneumococcal carriage rate was similar between children with NS and healthy children, children with NS have an increased risk for IPD and unvaccinated children are carriers for non-PCV13 serotypes.
